# Effects of Menopause in Women With Multiple Sclerosis: An Evidence-Based Review

**DOI:** 10.3389/fneur.2021.554375

**Published:** 2021-03-19

**Authors:** Riley Bove, Annette Okai, Maria Houtchens, Birte Elias-Hamp, Alessandra Lugaresi, Kerstin Hellwig, Eva Kubala Havrdová

**Affiliations:** ^1^Department of Neurology, UCSF Weill Institute for Neurosciences, University of California, San Francisco, San Francisco, CA, United States; ^2^Multiple Sclerosis Treatment Center of Dallas, Dallas, TX, United States; ^3^Partners Multiple Sclerosis Center, Brigham and Women's Hospital, Boston, MA, United States; ^4^Neurological Private Practice, Institute of Neuroimmunology and Multiple Sclerosis, Hamburg, Germany; ^5^IRCCS Istituto delle Scienze Neurologiche di Bologna, Bologna, Italy; ^6^Dipartimento di Scienze Biomediche e Neuromotorie, Università di Bologna, Bologna, Italy; ^7^Department of Neurology, Ruhr University Bochum and St. Josef-Hospital, Bochum, Germany; ^8^Department of Neurology and Center of Clinical Neuroscience, First Medical Faculty, General University Hospital, Charles University, Prague, Czechia

**Keywords:** multiple sclerosis, menopause, hormone therapy, best practices, fatigue, cognition

## Abstract

Over two thirds of all individuals who develop multiple sclerosis (MS) will be women prior to the age of menopause. Further, an estimated 30% of the current MS population consists of peri- or postmenopausal women. The presence of MS does not appear to influence age of menopausal onset. In clinical practice, symptoms of MS and menopause can frequently overlap, including disturbances in cognition, mood, sleep, and bladder function, which can create challenges in ascertaining the likely cause of symptoms to be treated. A holistic and comprehensive approach to address these common physical and psychological changes is often suggested to patients during menopause. Although some studies have suggested that women with MS experience reduced relapse rates and increased disability progression post menopause, the data are not consistent enough for firm conclusions to be drawn. Mechanisms through which postmenopausal women with MS may experience disability progression include neuroinflammation and neurodegeneration from age-associated phenomena such as immunosenescence and inflammaging. Additional effects are likely to result from reduced levels of estrogen, which affects MS disease course. Following early retrospective studies of women with MS receiving steroid hormones, more recent interventional trials of exogenous hormone use, albeit as oral contraceptive, have provided some indications of potential benefit on MS outcomes. This review summarizes current research on the effects of menopause in women with MS, including the psychological impact and symptoms of menopause on disease worsening, and the treatment options. Finally, we highlight the need for more inclusion of MS patients from underrepresented racial and geographic groups in clinical trials, including among menopausal women.

## Introduction

Multiple sclerosis (MS) is a chronic, immune-mediated, inflammatory, demyelinating disease of the central nervous system (1), with a female-to-male incidence rate ratio of 3:1 (2). Since MS is typically diagnosed in young adulthood (3), a majority of women living with MS will undergo menopause after MS diagnosis. However, rates of late-onset MS have also increased, particularly among women (4). Further, since the advent of disease-modifying therapies (DMTs) to treat MS, the life expectancy and median age of patients living with MS has increased (3), thereby necessitating awareness among clinicians of the changing needs associated with older patients.

For both women and men, increasing age is associated with changes in the MS course, notably a switch from a predominantly relapsing-remitting course to progressive phenotypes with greater disability accumulation (5). The symptoms of MS in older patients may be further impacted not only from the effects of somatic aging but also from the effects of other neurodegenerative diseases that are typical in older age (3). In addition, the onset of menopause in women presents further challenges for the management of MS since some symptoms experienced during this life transition, such as cognitive impairment, depression and anxiety, sleep disturbance and fatigue, and bladder impairment, can overlap with those of MS (6).

Changes in the levels of sex steroid hormones in women over time have long been postulated to affect the MS course, for example based on the well-established observation of reduced relapse rates in the third trimester of pregnancy, followed by a rebound postpartum (7). Some preclinical data suggest that exogenous hormones could impact disease course/severity *via* effects on neuroprotection and inflammation (8–10). Although much of the existing evidence on whether estrogen could aid in alleviating the effects of MS is from studies of women receiving oral contraceptives (11–13), an early retrospective study suggested a beneficial effect of hormone therapy (HT) in menopause (14).

In this review, we summarize the evidence on whether menopause has effects on MS symptoms and disease outcomes, additional to those effects anticipated with advancing age, and whether intervention with HT could improve quality of life and impact the MS disease course in postmenopausal women. We then provide recommendations for general management of these patients and for future study.

## Biology of Menopause and Functioning in MS

Menopause entails a number of physiological changes that affect women with MS through at least three physiological mechanisms: reproductive, immunological, and neurological. The onset of natural menopause in the general population typically occurs in the sixth decade of life; typically progesterone levels begin to decrease during the 30 s, whereas estrogen levels decline after a peak around the late 40 s (15). Anti-Müllerian hormone (AMH) is a key biomarker of ovarian aging, reflecting ovarian follicular reserve; levels generally peak around 25 years of age and gradually taper to undetectable by the time of menopause (16, 17).

The median age of natural menopause observed in women with MS is around 51 years (18, 19), aligning with that in the general population (20). Since AMH levels can give a more precise measure of ovarian reserve, they have also been investigated in case-control studies of women with MS. A study of women of reproductive age (*N* = 134) reported lower mean AMH levels in patients with MS (2.47 ± 0.26 ng/ml) compared with healthy controls (3.34 ± 0.34 ng/ml; *p* < 0.04) (21); in contrast, a larger study (*N* = 592) with a broader age range (22–65 years) found no difference in AMH levels by MS status (0.98-fold difference [95% CI, 0.69–1.37]; *p* = 0.87) (22). Therefore, it is not clear whether, overall, ovarian function is influenced by MS status.

Although several studies have evaluated the effect of menopause on aspects of MS disease course, including relapse rates, disability progression, and patient-reported outcomes, data are inconclusive. Over 10 years in a longitudinal study assessing disability progression in women with MS (*N* = 124), mean Expanded Disability Status Scale (EDSS) score increased from before to after menopause by 0.08 points (*p* = 0.02) (19). This finding was supported by a study of 148 women with MS that reported a greater mean EDSS score increase at an average of 3.5 years after menopause (0.4-point increase) compared with 3.5 years before menopause (0.2-point increase; *p* < 0.001); in contrast, annualized relapse rate (ARR) decreased from before to after menopause (0.21 vs. 0.13; *p* = 0.005) (23). Additionally, in an online reproductive history survey of 513 patients with MS, women who underwent surgical menopause had greater patient-reported disease severity as assessed by the MS Rating Scale (mean [SD] score: 13.1 [5.4]) compared with premenopausal women (mean [SD] score: 8.9 [5.5]; *p* < 0.0001 between groups); mean (SD) score for natural menopause was 9.6 (5.1) (18). A smaller retrospective study of 37 women with a diagnosis of MS prior to menopause supported declining relapse rate within 5 years after menopause (ARR: 0.08 vs. 0.37 before menopause; *p* < 0.001), but not increased disability, with no change reported after menopause for either EDSS progression rate (0.13-point increase per year both before and after menopause; *p* = 0.94) or frequency of EDSS progression events (37.8 vs. 48.6%; *p* = 0.42) (24). In a recent systematic review, when the data from the Baroncini et al. (23) and Ladeira et al. (24) studies were assessed in aggregate, no overall difference between relapse rates before and after menopause was found (risk ratio: 1.21; 95% CI, 0.91–1.61; *p* = 0.218) (25).

An important factor likely to contribute to age-related MS disability progression is immunosenescence, which affects both the adaptive and innate arms of the immune system (5, 26). Studies of peripheral biomarkers of immunosenescence have indicated that patients with MS may display a particular type of immunosenescence that can have a premature onset (27). Furthermore, menopause-related declines in sex hormone levels may contribute to a reproductive senescence (28) that would add to the effects of more general age-related immunosenescence (29, 30). For example, some data suggest declining ovarian reserve is associated with neurodegeneration in MS patients, as evidenced by brain volume loss. In a cohort study of 412 women with MS, 10-fold lower AMH levels were linked with accelerated reduction of gray matter volume in both a cross-sectional analysis (change in cortical gray matter: −7.44 mm^3^; *p* = 0.041) and a longitudinal analysis with up to 10 years of follow-up (change in cortical gray matter: −4.55 mm^3^; *p* = 0.062) and increased EDSS score (cross-sectional analysis: 0.43-point increase [*p* = 0.003]; longitudinal analysis: 0.27-point increase [*p* = 0.006]) (22). However, it is possible that declining ovarian function is colinear with declining brain function, rather than contributory to it; further, since such studies cannot include comparator cohorts of men, the relative contributions of general aging and declining reproductive function to these findings have not been fully elucidated (31).

In addition to immunosenescence, another phenomenon that occurs with increasing age is a general, low-level increase in the production of proinflammatory cytokines (5). By promoting neuroinflammation, this process of “inflammaging” is thought to increase the risk of cognitive impairment in MS patients (5), which is one of the key symptoms that can overlap between menopause and MS (6).

## Clinical Assessment and Care of Women With MS

### Signs and Symptoms

Among MS patients, cognitive decline affects up to 65% of patients (32), and may include changes in memory, attention, executive function, information processing, and processing speed (33). Cognitive impairment in MS is of particular importance to menopausal patients as it has been consistently shown to worsen with older age (34, 35), despite no consistent evidence in the literature for different frequencies of cognitive problems between men and women with MS (34).

Relative to the general population, the prevalence of anxiety and depression is higher in patients with MS (36, 37), up to half of whom experience depression (38). In patients with MS, no consistent correlation has been established between increasing risk of depression and older age or female sex; indeed, almost half of the variance associated with rates of depression in MS is thought to reflect structural brain changes (5, 39). Nonetheless, certain factors may impart a particular vulnerability on the psychological well-being of menopausal women with MS. In some women undergoing the transition to menopause, declining estrogen levels may increase the risk of depression (40). Furthermore, given that many patients with MS develop a more progressive course after the age of 45 years (41), women may be grappling with not only the physical but also the psychological implications of this diagnosis at the time of menopause.

Of particular relevance to depression and anxiety, sleep disturbances are a common occurrence among healthy women in perimenopause (42). The prevalence of sleep dysfunction in patients with MS has been reported to be over 50%, which is substantially higher than in the general population, and it is more common in women with MS than in their male counterparts (43). Poor sleep patterns in patients with MS can result in cognitive impairments and changes in mood, with the link between poor sleep quality and depression and anxiety being particularly strong in women with MS (44). These disruptions in sleep have the potential to negatively impact other areas, such as decreasing quality of life and exacerbating comorbidities (43, 45). In particular, attentive management of factors such as sleep interruptions and depression may aid in reducing secondary fatigue, which may be modifiable in MS patients, in contrast to the primary fatigue thought to be caused by demyelination, inflammation, and axonal damage (46).

Urinary and sexual dysfunction are common occurrences in both menopausal women and in women with MS (47–50). Recent studies have investigated the potential influence of menopausal status on sexual dysfunction in women with MS. In a study of 248 women with MS, the rate of sexual dysfunction as measured by the Female Sexual Function Inventory (FSFI) was higher in postmenopausal (72/96 [75%]) than in premenopausal women (88/152 [58%]), albeit with no significant correlation found between menopausal status and FSFI subscales (51). In another study of 306 women, the proportion of postmenopausal women with MS with sexual dysfunction, as defined by FSFI score <26.55 and Female Sexual Distress Scale score >15, was 20/40 patients (50.0%). This proportion was higher than that among premenopausal women with MS (30/79 [37.9%]), but with the difference not reaching statistical significance (*p* = 0.24), and significantly higher than that for postmenopausal women without MS (16/57 [28.1%]; *p* = 0.03) (52).

In all women, the risk of osteoporosis and related fractures increases post menopause (53, 54). However, osteoporosis is more common in patients with MS compared with healthy populations; bone loss starts early during MS disease course, and increases as the disease progresses (55, 56). Moreover, there is evidence showing that chronic use of glucocorticosteroids reduces bone formation and is a risk factor for osteoporotic fractures (57), although data from studies in MS patients are conflicting (58–60). In a case-control study examining the association between MS and likelihood of developing osteopenia or osteoporosis, a total of 91 men (*n* = 45) and women (*n* = 46) with MS (mean [SD] age: 52.0 [10.3] years) had a total body bone mineral density of 1.12 g/cm^2^ and T-score of −0.6, indicating total bone density was not in the range of osteopenia or osteoporosis according to the World Health Organization classification. However, patients with MS in this analysis had bone density in the lumbar spine (bone density: 1.07 g/cm^2^; T-score: −1.09) and the left femoral hip (bone density: 0.69–0.86 g/cm^2^; T-score: −1.43 to −1.56) indicative of osteopenia, suggesting bone loss may be more prominent in certain areas of the body among MS patients (56). The North American Menopause Society recommends bone mineral density be tested in postmenopausal women who are at a higher risk of osteoporosis due to medical conditions, such as MS, and provides guidance for pharmaceutical management strategies (61).

Additionally, the risk of hypertension or cardiovascular comorbidities increases with age in both the general population and in patients with MS (62). In studies based on the North American Research Committee on Multiple Sclerosis Registry (*N* = 8,983), the presence of vascular comorbidities at MS diagnosis was associated with more severe disability at the time of MS diagnosis (odds ratio for moderate vs. mild disability: 1.51, 95% CI 1.12–2.05) (63), as well as higher risk of MS-related disability progression (hazard ratio per vascular condition for early gait disability: 1.51, 95% CI 1.41–1.61) (64). Cardiovascular comorbidities may also influence the relative benefits and risks of various DMTs. For example, secondary hypertension is linked to certain categories of DMTs including sphingosine-1-phosphate (S1P) inhibitors (e.g., fingolimod) (65–67) and teriflunomide.

### Management of Menopausal Symptoms

Approaches for the management of menopausal symptoms include HT, herbal supplements available over the counter (e.g., soy and black cohosh), and off-label use of selective serotonin reuptake inhibitors (SSRIs) and anticonvulsants. HT includes estrogen therapy or combined estrogen-progestogen therapy, administered in both systemic (e.g., oral) and local (e.g., vaginal cream) formulations.

In MS, to date, little is known about the effect of HT on disease course. Research into the possible protective effects of HT on MS symptoms and overall well-being during the menopausal transition is of significant clinical importance. Few women observed in modern MS cohorts receive HT. For example, only 18.2% of the women observed in the Comprehensive Longitudinal Investigation of MS at the Brigham and Women's Hospital (CLIMB) study had used estrogen HT either alone or in combination with progesterone within 5 years of menopause (19). In an analysis of MS patients in the Nurses' Health Study (*N* = 248), a historical observational cohort, HT at the time of menopause was associated with better physical quality of life, as measured by the 10-item physical functioning assessment (PF10) subscale of the 36-Item Short Form Health Survey (*p* = 0.004) (68). However, this finding may not have reflected causality and could be explained by the fact that women with better physical quality of life are more likely to receive general preventative care (69, 70), including possible HT at the time that the cohort underwent menopause. Results from two interventional clinical trials assessing systemic exogenous estrogens in premenopausal women with MS are available. In a study of 164 women with relapsing-remitting MS (RRMS) aged 18–50 years who were receiving glatiramer acetate 20 mg, estriol treatment reduced the ARR over 2 years compared with placebo (adjusted rate ratio 0.63, 95% CI 0.37–1.05; *p* = 0.077) (71). Over 2 years in a randomized controlled trial of 150 women with RRMS aged 18–45 years, patients receiving interferon beta-1a (IFNB-1a) combined with ethinylestradiol 40 μg and desogestrel 125 μg as oral contraceptive showed a 26.5% reduction (*p* = 0.04) in the cumulative number of combined unique active lesions on brain magnetic resonance imaging (MRI), as well as a higher likelihood to be free from gadolinium-enhancing lesions (*p* = 0.03), compared with IFNB-1a alone (72). A *post-hoc* analysis of this study additionally reported a lower risk of cognitive impairment in the group who received ethinylestradiol 40 μg and desogestrel 125 μg combined with IFNB-1a, but also an increased risk of sexual dysfunction (*p* = 0.03 vs. IFNB-1a alone for both findings) (73). Results from a recently completed pilot trial in menopausal women are anticipated (NCT02710214).

In the general population, because of its superior efficacy, as well as some side effects associated with the other therapeutic options, HT is often the preferred therapy for treating menopausal symptoms. In a 2017 consensus statement, the North American Menopause Society concluded that HT remains the most effective treatment for menopausal vasomotor and genitourinary symptoms and may prevent bone loss and fracture (74). However, there are some risks, such as breast cancer, when progestogens are given concurrently with estrogens, as well as venous thrombosis despite a reduced risk of cardiovascular disease overall. Current interpretations of the results of the Women's Health Initiative do not support giving systemic estrogen therapy or estrogen-progestin therapy to prevent chronic diseases, including coronary heart disease and invasive breast cancer, even in young women, in the general population (75). With respect to neurological function, some observational studies have reported better cognition when HT was started within a 5-year window of the final menstrual period (76). However, interventional studies have yielded a more mixed picture (77, 78). The Women's Health Initiative administered HT to women well beyond their menopausal transition, and reported increased risk of stroke and dementia (79). Recent re-analyses of women within a narrower postmenopausal window have been more reassuring, and there are no cognitive contraindications to HT for menopausal women experiencing vasomotor symptoms (74).

In women for whom HT is contraindicated (e.g., prior breast cancer or personal preference), there are other treatment options for menopausal symptoms. For example, for the treatment of vasomotor symptoms, SSRIs, norepinephrine reuptake inhibitors, and anticonvulsants have demonstrated greater efficacy vs. placebo (80–83). Although side effects with SSRIs are typically short lived, those experienced with anticonvulsants may be more severe and are therefore a limiting factor in their use within the perimenopausal/menopausal population (42, 84). Trials of herbal remedies have shown no significant effects on vasomotor symptoms compared with placebo (85). As an approach for bone density preservation, the use of alendronate might be recommended. In the overlap of bladder symptoms due to menopause and MS, intravesical botulinum toxin and pelvic floor therapy may be effective (86), especially in women in whom HT should be avoided and who might also have contraindication to anticholinergics due to the potential to worsen cognitive function. Finally, addressing sleep disturbances early on, may aid in avoiding subsequent chronic sleep problems. When introducing symptomatic therapies, it is important to consider overall safety and any possible pharmacological side effects or interactions. For example, lower doses of sleeping agents such as zolpidem are recommended in women than in men.

### Regional and Societal Differences

Research in MS, including the effects of menopause, on symptoms, neurological function, and quality of life are often carried out in White women from Western cultures. The extent to which findings on menopause can be translated to women from other racial and ethnic groups, as well as different countries or continents, is unknown (87, 88). Lock and Kaufert reported lower rates of menopause-associated symptoms, including hot flashes, sleep disturbances, and low mood, in women from Japan compared with women from the United States and Canada, and comparable rates to those reported in China and Thailand. Additionally, in these populations, the postmenopausal period could entail different risks for chronic diseases (88). Results from the Study of Women's Health Across the Nation, which included 14,906 middle-aged women from across the United States, showed increased psychosomatic symptoms reported in White women and greater vasomotor symptoms reported in African American women vs. other racial and ethnic populations (87). These findings highlight the need to consider not only genetic, but also physiological, social, and cultural factors in studies of menopause and MS.

### Recommended Screenings for Menopausal MS Patients

Women with MS undergoing the menopausal transition may experience symptoms from menopause-associated physiological changes, MS disability progression, and age-associated comorbidities simultaneously, making it important to proactively consider multifactorial causes of worsening symptoms or function at menopause.

Early and appropriate screenings for comorbidities associated with menopause and MS are, therefore, recommended. Screenings should include, but are not limited to, blood pressure, cancer, and bone density screenings, including assessments for confounding behavioral factors such as smoking. Neglected cancer screenings in patients with MS have the potential to impact mortality, and effort to rule out cancer should therefore be undertaken whenever symptoms suggest the possibility of causality outside the general scope of MS (70). Similarly, evaluating for and preventing osteoporosis through appropriate bone density screenings could partially reduce the increased bone fracture risk in MS patients (53, 54), who are also at risk of falls. As smoking can differentially impact pre- vs. postmenopausal women, it is important to determine whether such a confounding factor may be contributing to loss of bone density. Whenever possible, smoking cessation should be encouraged as a means of reducing risk for bone fracture (61).

## Author Recommendations

Table 1 provides a summary of recommendations and features for neurologists and other health care professionals for the care of menopausal women with MS.

**Table 1:** Summary of author recommendations for neurologists and other health care professionals.

**Topic**	**Recommendation**
**Reproductive**	
Management of symptoms	HT can alleviate vasomotor and other symptoms associated with menopause. HT with a combination of estrogen and progestin is recommended to decrease endometrial and breast cancer risk in postmenopausal women with or without MS (54, 89–91).
**Bladder symptoms**	
	Consider intravesical botulinum toxin and pelvic floor therapy as options for symptomatic treatment of bladder impairment, especially in women for whom HT or anticholinergics are contraindicated (86). Comprehensive evaluation of bladder function including stress and urge incontinence as well as retention.
**Exogenous hormone use**	Exogenous hormones could impact disease course/severity *via* effects on neuroprotection and inflammation, although research is limited, specifically in an aging population (11–13, 92, 93).
**Immunological**	
Infections	Monitor for increased risk of infections, regardless of whether patients are treated with DMTs (94).
**Comprehensive care**	
Cancer	Ensure appropriate cancer screening per guidelines, e.g., mammogram, cervical cancer screening, colonoscopy (95). Women with disabilities, including MS, are less likely to get screening (possibly due to clinician biases and more burdensome medical care) (70).
Coordinating care with neurologists and other HCPs	Collaborate and communicate with the patient's primary care provider and other HCPs caring for the patient.
**Neurological**	
Cognitive impairment	Cognitive evaluation and, if warranted, rehabilitation to improve upon the cognitive domains impaired in MS (96). To date, there are no proven benefits of HT on cognition (76, 78).
**Psychological**	
Psychotherapy	Use comprehensive treatment approaches to manage symptoms associated with psychological changes during menopause (97).
**Regional and societal differences in the experience of menopause**	
Menopausal status	Consider differences based on racial, ethnic, cultural, or geographical factors, including the age of MS onset and the different experiences of menopausal symptoms (87, 88, 98).

*DMT, disease-modifying therapy; EDSS, Expanded Disability Status Scale; HCP, health care professional; HT, hormone therapy; MRI, magnetic resonance imaging; MS, multiple sclerosis*.

## Conclusion

For professionals to effectively manage and care for the female MS population regardless of age, more research is required to disentangle the effects of menopause on symptoms and the disease course in patients with MS. Further longitudinal studies on MS disease activity in diverse populations of women with MS are needed. Of specific interest will be more randomized controlled clinical trials to investigate the possible protective effect of HT on women with MS, and to investigate the benefit-to-risk ratio in this population, which may differ from the general population. In addition, available information regarding DMTs in postmenopausal women with MS is currently limited, as clinical trials in MS often restrict enrollment to those aged ≤ 50 or ≤ 55 years. To provide for evidence-based decisions in an older patient population, trial designs should aim to include patients who are aged >50 years. Enhanced understanding of the relationship between sex steroids, menopause and immunosenescence may also provide new opportunities for management of MS in women. Interventions and treatments, as well as guidance and support, are needed for patients who may be particularly vulnerable to physiological and psychological decline at this time point in their lives, and beyond.

## Author Contributions

RB, AO, MH, BE-H, AL, KH, and EKH provided conception, critical review and revision, and final approval of the manuscript for submission. All authors contributed to the article and approved the submitted version.

## Conflict of Interest

RB reports consultancy fees from Alexion, Biogen, EMD Serono, Novartis, Roche Genentech, and Sanofi Genzyme; and research support from Akili Interactive and Roche Genentech. AO reports consulting fees from Biogen, Bristol Myers Squibb, EMD Serono, Roche Genentech, and Sanofi Genzyme; and research support from Alexion, Novartis, Roche Genentech, and Sanofi Genzyme. MH reports consulting fees from Biogen, Genentech, Genzyme, Serono, and Teva; and research and support from Biogen, Genzyme, and Serono. BE-H reports research support, consultancy fees, speaker fees, and personal compensation for activities with Almirall, Bayer HealthCare, Biogen, MedDay, Merck Serono, Novartis, Roche, Sanofi Genzyme, and Teva. AL has served as a Biogen, Merck Serono, Mylan, Novartis, Roche, Sanofi Genzyme, and Teva Advisory Board Member. She received congress and travel/accommodation expense compensations or speaker honoraria from Biogen, Merck, Mylan, Novartis, Sanofi Genzyme, Teva, and Fondazione Italiana Sclerosi Multipla (FISM). Her institutions received research grants from Novartis. KH reports consultancy fees, speaker fees, and research support from Bayer, Biogen, Merck Serono, Novartis, Roche, Sanofi Genzyme, and Teva. EKH reports speaker honoraria and research grant support from Actelion, Biogen, Celgene, Merck Serono, Novartis, Sanofi Genzyme, and Teva; compensation for advisory board activities from Actelion, Biogen, Celgene, Genzyme, and Novartis; and support from the Czech Ministry of Education, Project PROGRES Q27/LF1.

## Abbreviations

AMH: anti-Müllerian hormone
ARR: annualized relapse rate
DMT: disease-modifying therapy
EDSS: Expanded Disability Status Scale
FSFI: Female Sexual Function Inventory
HT: hormone therapy
IFNB-1a: interferon beta-1a
MS: multiple sclerosis
RRMS: relapsing-remitting MS
SSRI: selective serotonin reuptake inhibitor.
